# An Integral Recognition and Signaling for Electrochemical Assay of Protein Kinase Activity and Inhibitor by Reduced Graphene Oxide-Polydopamine-Silver Nanoparticle-Ti^4+^ Nanocomposite

**DOI:** 10.3389/fbioe.2020.603083

**Published:** 2020-11-13

**Authors:** Jialong Wang, Xueqian Liu, Chao Wang, Dengren Liu, Fang Li, Li Wang, Shufeng Liu

**Affiliations:** ^1^Key Laboratory of Optic-Electric Sensing and Analytical Chemistry for Life Science, Ministry of Education, College of Chemistry and Molecular Engineering, Qingdao University of Science and Technology, Qingdao, China; ^2^College of Marine Science and Biological Engineering, Qingdao University of Science and Technology, Qingdao, China

**Keywords:** protein kinase, electrochemical biosensor, reduced graphene oxide, polydopamine, silver nanoparticle

## Abstract

A novel electrochemical biosensing method for protein kinase (PKA) activity was demonstrated by using a reduced graphene oxide-polydopamine-silver nanoparticle-Ti^4+^ (rGO-PDA-AgNPs-Ti^4+^) nanocomposite. The obtained nanocomposite possessed an integral capability for phosphopeptide recognition and signal readout. The polydopamine modified reduced graphene oxide (rGO-PDA) was firstly prepared based on a self-polymerization method of dopamine. The silver ions were adsorbed onto polydopamine (PDA) layer and directly reduced into silver nanoparticles (AgNPs), which was used for electrochemical signal reporting. Then, the Ti^4+^ cations were attached onto the PDA layer for phosphopeptide recognition according to the strong coordination ability of PDA with Ti^4+^ and phosphate group. The prepared rGO-PDA-AgNPs-Ti^4+^ nanocomposites were characterized with different methods. The developed rGO-PDA-AgNPs-Ti^4+^ nanocomposites were then employed for electrochemical analysis of PKA-catalyzed kemptide phosphorylation. The sensitive detection toward PKA activity was realized with an experimental detection limit of about 0.01 U/mL. It may be also extended for the inhibitor evaluation. Thus, it provided a facile and sensitive means for electrochemical analysis of PKA activity and inhibitor screening.

## Introduction

Protein kinase (PKA)-catalyzed protein phosphorylation is an important protein post-translational modification event that plays a crucial role in cellular signaling pathways (Johnson and Lewis, 2001; Manning et al., 2002; Tarrant and Cole, 2009). The aberrant phosphorylation status and PKA activity is closely associated with some clinical diseases (Skorski, 2002; Stergiopoulos and Stratakis, 2003; Hardie, 2014). Protein kinases have been also extensively applied as therapeutic targets in drug discovery and design (Noble et al., 2004; Cohen and Alessi, 2013). Thus, identification of PKA activity would be important for understanding of corresponding biological processes, disease diagnosis, and drug discovery.

Till now, PKA activity has been assayed by using different techniques such as electrochemistry, fluorescence, colorimetry, photoelectrochemistry, electrochemiluminescence, etc., (Houseman et al., 2002; Wang et al., 2006; Herbst et al., 2011; Liu et al., 2016; Li et al., 2017; Liu J. et al., 2017; He et al., 2018; Harrington et al., 2019). Electrochemical method is especially intriguing for biosensor fabrication owing to its prominent advantages for example simple instrumentation, operational flexibility, ease of miniaturization and good sensitivity. The detection principle for PKA is usually based on the following two types. The labeling technique is the mostly used type for PKA activity evaluation. For example, the radioactive, electroactive, fluorescent, biotin, or thiol labels could be introduced into the kemptide substrate during PKA catalysis process by using the corresponding ATP analogs as alternatives (Xu S. et al., 2010; Freeman et al., 2010; Martic et al., 2011; Gu et al., 2018; Tian et al., 2019). Then, the PKA activity could be assayed by different methods. Another commonly used type for PKA assay is based on the phosphorylation-specific recognition elements. These include antibody, metal complexes, metal ions, and nanomaterials (Wang et al., 2011; Lowe et al., 2012; Liu et al., 2014; Zhou et al., 2015; Jia et al., 2020). After PKA catalyzed kemptide phosphorylation, the phosphate group could be specifically recognized by these recognition elements for further signal response. Especially, some high valence metal ions or their complex for example Zr^4+^ and Ti^4+^ are intriguing for the enrichment of the phosphorylated peptide owing to their strong multi-coordination ability with the phosphate group (Monot et al., 2008; Pang et al., 2011; Yan et al., 2013; Salimi et al., 2017). They are also explored to fabricate various PKA biosensors. The DNA, polymer, or nanomaterials could be further introduced as the signal amplification systems for the sensitive detection of PKA activity (Wang et al., 2014; Chen et al., 2018; Hu et al., 2019). But most of strategies required the complicate operation procedures for signal amplification. Thus, development of the facile, reliable and highly sensitive electrochemical biosensor for PKA activity is still highly desirable.

In this text, a facile and sensitive electrochemical strategy for PKA activity assay was developed by using a reduced graphene oxide-polydopamine-silver nanoparticle-Ti^4+^ (rGO-PDA-AgNPs-Ti^4+^) nanocomposite as an integral sensing platform for phosphopeptide recognition, signal amplification, and readout. Dopamine could be self-polymerization in the alkaline solution into the hydrophilic polydopamine (PDA) that shows the strong adhesion ability toward almost all substrates (Lee et al., 2007; Kang et al., 2009). In the case of GO as the substrate, the GO could be reduced into rGO by the DA during the self-polymerization process of DA, resulting in the rGO-PDA nanocomposite. The PDA coating possessed an excellent environmental stability, good biocompatibility and water dispersibility (Tang et al., 2019). Owing to the presence of catechol hydroxyl-groups, the PDA coating provided binding sites for silver ions, which were *in-situ* reduced into silver nanoparticles (AgNPs). Then, the Ti^4+^ cations were attached onto the PDA layer based on the chelation of Ti^4+^ cations with the diol groups of PDA to obtain the rGO-PDA-AgNPs-Ti^4+^ nanocomposite. The Ti^4+^ was committed for phosphopeptide recognition. The AgNPs was used for the direct readout of electrochemical signal. The obtained rGO-PDA-AgNPs-Ti^4+^ nanocomposites were then used for PKA biosensor fabrication. The immobilized kemptide on the electrode could be phosphorylated by PKA and the phosphorylated kemptide was captured by the rGO-PDA-AgNPs-Ti^4+^ nanocomposite for electrochemical responses related with PKA activity.

## Experimental Section

### Chemicals and Reagents

Graphene oxide (GO) was obtained from Shanghai Tanyuanhuigu Co., Ltd. (Shanghai, China). Exonuclease III (Exo III) and cAMP-dependent protein kinase (PKA) were obtained from New England Biolabs Inc. (Ipswich, MA, United States). Cysteine-terminated kemptide (LRRASLGGGGC) and ATP were purchased from Sangon Biotech Co., Ltd. (Shanghai, China). Thrombin, hemoglobin (Hb), bovine serum albumin (BSA) and fetal calf serum (FBS) were supplied by Dingguo Biotech Co., Ltd. (Beijing, China). Dopamine hydrochloride and N-[2-(p-bromocinnamylamino)ethyl]-5-isoquinolinesulfonamide dihydrochloride (H-89) were purchased from Aladdin Reagents Inc. (Shanghai, China). Tris-(hydroxymethyl)aminomethane (Tris), AgNO_3_ and Ti(SO_4_)_2_ were supplied by Sinopharm Chemical Reagent Co., Ltd. (Shanghai, China). Ultrapure water was always used and obtained from a Milli-Q water purification system (Millipore Corp., Bedford, MA, United States).

### Preparation of Reduced Graphene Oxide-Polydopamine Nanocomposite (rGO-PDA)

The reduced graphene oxide-polydopamine (rGO-PDA) composite was firstly obtained by a self -polymerization method of dopamine according to the previous literature (Xu L. Q. et al., 2010; Kang et al., 2011). Simply, graphite oxide (50 mg) was first dissolved into 100 mL of Tris buffer (10 mM, pH 8.5). After sonication for 30 min, the dopamine hydrochloride (200 mg) was added and the mixture was kept for 12 h with vigorous stirring at room temperature. After centrifugation and washing with deionized water and ethanol for several times, the precipitate was vacuum-dried (60°C, 12 h) to obtain the rGO-PDA nanocomposite.

### Preparation of Reduced Graphene Oxide-Polydopamine-Silver Nanoparticle-Ti^4+^ (rGO-PDA-AgNPs-Ti^4+^) Nanocomposite

0.5 mL of 100 mM AgNO_3_ was added into 4.5 mL of 2 mg/mL rGO-PDA suspension and reacted for 3 h at room temperature. After that, the mixture was centrifuged and washed with deionized water to remove the unreacted Ag^+^. The rGO-PDA-AgNPs were obtained and diluted to the original volume. Then, 10 mM Ti(SO_4_)_2_ was added into the above rGO-PDA-AgNPs suspensions and stirred for 2 h at room temperature. The mixture was then centrifuged and washed with deionized water to remove the unreacted Ti^4+^. Then, the rGO-PDA-AgNPs-Ti^4+^ precipitates were diluted to the original volume to obtain the rGO-PDA-AgNPs-Ti^4+^ suspension.

### Kemptide Immobilization

The gold electrode with a 2 mm diameter was pretreated based on the reported method (Liu S. et al., 2017). The immobilization of kemptide was executed by incubating the gold electrode into 200 μM kemptide in 10 mM Tris-HCl buffer (pH 7.4, 0.2 M NaCl, 10 mM TCEP, 1 mM EDTA) for overnight (room temperature). After kemptide immobilization, the electrode was washed with 10 mM Tris-HCl buffer (pH 7.4, 0.1 M NaCl). Then, the electrode was incubated into 1 mM MCH for 60 min and washed with Tris-HCl buffer (pH 7.4, 0.1 M NaCl).

### PKA-Catalyzed Kemptide Phosphorylation and Recognition

The kemptide phosphorylation process was operated in 50 mM Tris-HCl buffer (pH 7.4, 20 mM MgCl_2_) that contained different concentrations of PKA and 100 μM ATP. After incubation at 37°C for 90 min, the electrode was washed with Tris-HCl buffer. Then, the phosphorylated electrode was incubated into the 50 μL above rGO-PDA-AgNPs-Ti^4+^ nanocomposite suspension for 1 h (room temperature) to accomplish the recognition of nanocomposites with the phosphorylated kemptide.

### Screening of PKA Inhibitor

Different concentrations of H-89 were firstly mixed into 50 mM Tris-HCl buffer (pH 7.4, 20 mM MgCl_2_) that contained 100 μM ATP and 10 U/mL PKA for 20 min at 37°C. The concentrations of H-89 in the mixed solution were 5, 10, 25, 50, 100, 200, and 500 nM, respectively. Then, the immobilized kemptide was incubated into the above mixed solution for 90 min at 37 °C. After washing by Tris-HCl buffer, the electrode was incubated into the 50 μL rGO-PDA-AgNPs-Ti^4+^ nanocomposite suspension for 1 h at room temperature and then electrochemical interrogation was conducted.

### Electrochemical Detection

Differential pulse voltammetry (DPV) and cyclic voltammetry (CV) characterizations for PKA activity was operated in 10 mM Tris-HCl (10 mM NaCl, pH 7.4). DPV was scanned from 0 to 0.4V with a pulse amplitude and width set at 50 mV and 50 ms, respectively. CV was scanned in the potential range between −0.5 and 0.5 V with a scan rate of 100 mV/s. The electrochemical impedance spectroscopy (EIS) characterization toward the biosensor fabrication process was conducted in 5 mM [Fe(CN)_6_]^3–/4–^ of 10 mM PBS buffer (pH 7.4, 1 M KCl). The applied frequency was in the range from 0.1 Hz to 10 K Hz.

### Apparatus

The morphology and structure were characterized by a transmission electron microscope (TEM, FEI Tecnai G20, United States) and a field emission scanning electron microscopy (SEM, JSM-7500F, JEOL, Japan). X-ray diffraction (XRD) patterns were obtained on a Bruker D8ADVANCE diffractometer (D8ADVANCE, Bruker, Germany) by the filtered Cu Kα radiation (λ = 1.5406 Å). UV-Vis spectroscopy was obtained by a UV-vis spectrophotometer (UV-2600 SHIMADZU, Japan). The electrochemical tests were conducted on a CHI 660D electrochemical workstation (Shanghai, China) with the use of a three-electrode system. The modified gold electrode, platinum wire and Ag/AgCl electrode were used as the working, auxiliary and reference electrodes, respectively.

## Results and Discussion

### The Sensing Principle for Protein Kinase Activity

**SCHEME 1:**
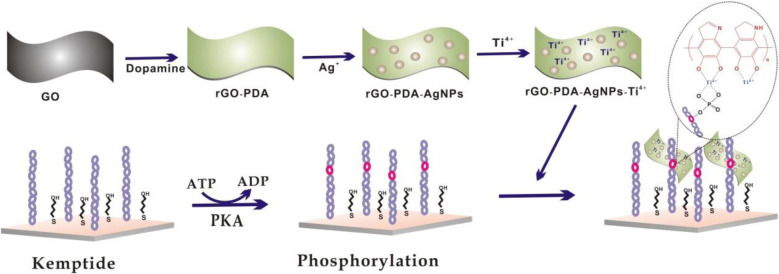
Schematic illustration of electrochemical biosensor for PKA activity assay based on an integral rGO-PDA-AgNPs-Ti^4+^ nanocomposite.

The overall synthesis procedure of rGO-PDA-AgNPs-Ti^4+^ nanocomposite and the detection principle for protein kinase activity was shown in (Scheme 1. The synthesis of rGO-PDA-AgNPs-Ti^4+^ nanocomposite was based on the following three steps. First, the PDA layer was coated onto GO surface via the self-polymerization of dopamine in alkaline condition. At the same time, GO could be reduced into rGO by the DA to acquire the rGO-PDA nanocomposite (Xu L. Q. et al., 2010; Kang et al., 2011). The PDA layer could adsorb silver ions and directly reduce them into AgNPs in needless of other reductants (Zhou et al., 2013; Lu et al., 2015). Thus, the rGO-PDA-AgNPs nanocomposite was obtained. Finally, the Ti^4+^ cations were attached onto the PDA layer based on the chelation of Ti^4+^ cations with the diol groups of PDA to obtain rGO-PDA-AgNPs-Ti^4+^ nanocomposite (Yan et al., 2013). Then, the rGO-PDA-AgNPs-Ti^4+^ nanocomposites were used for electrochemical investigation of PKA-catalyzed kemptide phosphorylation. The cysteine-terminated kemptide was assembled on the electrode via the covalent interaction of thiol group of cysteine with Au. The kemptide phosphorylation was accomplished by the PKA-catalyzed transfer of the γ-phosphate group of ATP into the serine of kemptide. The phosphorylated kemptide was then directly captured by the rGO-PDA-AgNPs-Ti^4+^ nanocomposite based on the multi-coordinative interaction between Ti^4+^ and the phosphate group, generating an electrochemical response toward PKA activity.

### Experimental Characterization of Nanocomposites

**FIGURE 1:**
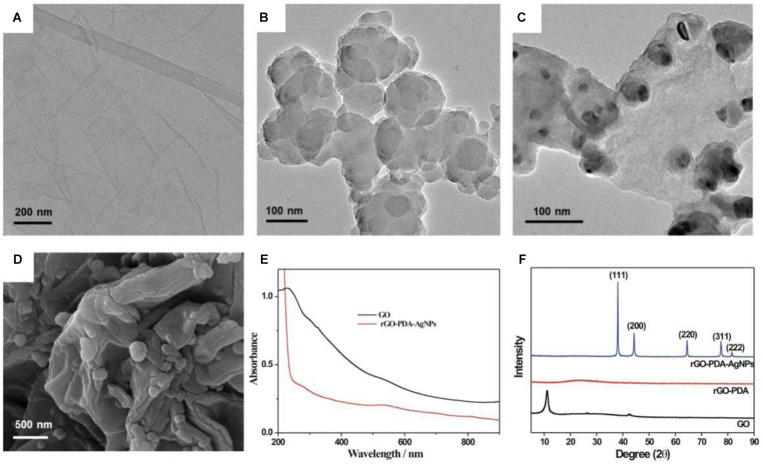
TEM images for GO **(A)**, rGO-PDA **(B)** and rGO-PDA-AgNPs **(C)**. **(D)** SEM image for rGO-PDA-AgNPs. **(E)** UV-vis spectroscopy of GO (black) and rGO-PDA-AgNPs (red). **(F)** XRD patterns of GO (black), rGO-PDA (red) and rGO-PDA-AgNPs (blue).

The morphology characterizations toward GO and rGO-PDA and rGO-PDA-AgNPs were shown in Figures 1A–D. The TEM image of GO appeared transparent and folded over the edges (Figure 1A). After PDA modification, the surface of rGO-PDA became smooth with the wrinkles disappeared. Also the aggregation for the rGO-PDA was observed (Figure 1B). This indicated the formation of PDA layer onto rGO surface by self-polymerization of dopamine. As for rGO-PDA-AgNPs, the AgNPs could be seen to be distributed onto the surface of rGO-PDA (Figures 1C,D).

The GO and the resulting rGO-PDA-AgNPs were also characterized by UV-vis absorption spectroscopy (Figure 1E). The GO displayed a typical absorption located at 230 nm and a shoulder absorption at about 300 nm, which were attributed to the π→π^∗^ transition (aromatic C-C bonds) and n→π^∗^ transition (carbonyl groups), respectively (Das et al., 2014; Hu et al., 2014). After modification with PDA and AgNPs loading, GO was reduced into rGO by dopamine. At about 278 nm, a new peak appeared, which was a characteristic absorption related with catechols in polydopamine. Also, there was a clear peak at about 540 nm. This was characteristic of AgNPs due to surface plasmon absorption, implied the formation of rGO-PDA-AgNPs.

The formation of rGO-PDA-AgNPs was further characterized by XRD (Figure 1F). A diffraction peak located at 2*θ* = 11° could be attributed to the (001) reflection of GO. For the rGO-PDA, it could be seen that the diffraction peak at 11° disappeared, indicating the reduction of GO into rGO. The XRD pattern of rGO-PDA-AgNPs showed the distinct diffraction peaks at 38.2°, 44.3°, 64.4°, 77.5°, and 81.5° respectively, related with the reflections from (111), (200), (220), and (311) planes of the metal Ag (JCPDS Card No. 04-0783). This indicated the face-centered cubic structure of the AgNPs onto rGO-PDA surface.

**FIGURE 2:**
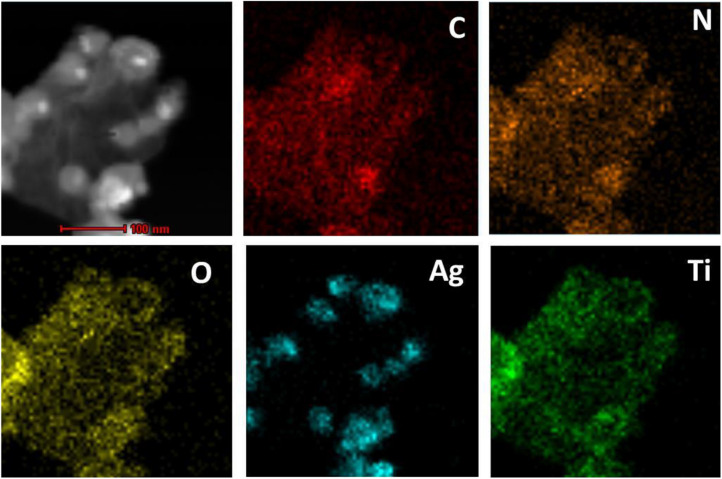
Corresponding elemental mapping images of C, N, O, Ag, and Ti.

The obtained rGO-PDA-AgNPs-Ti^4+^ nanocomposites were also characterized by the elemental mapping images (Figure 2). It could be seen that the elements of C, N, O, Ag, and Ti was rationally distributed throughout the nanocomposite. Especially, the Ti and Ag elements occupied the complementary locations, suggesting the competitive coordination of the Ag^+^ and Ti^4+^ with the diol groups of PDA. The EDX spectra further disclosed the elements and contents of C, N, O, Ag, and Ti in rGO-PDA-AgNPs-Ti^4+^ nanocomposite (Supplementary Figure S1).

### Electrochemical Detection Feasibility Toward PKA Activity

**FIGURE 3:**
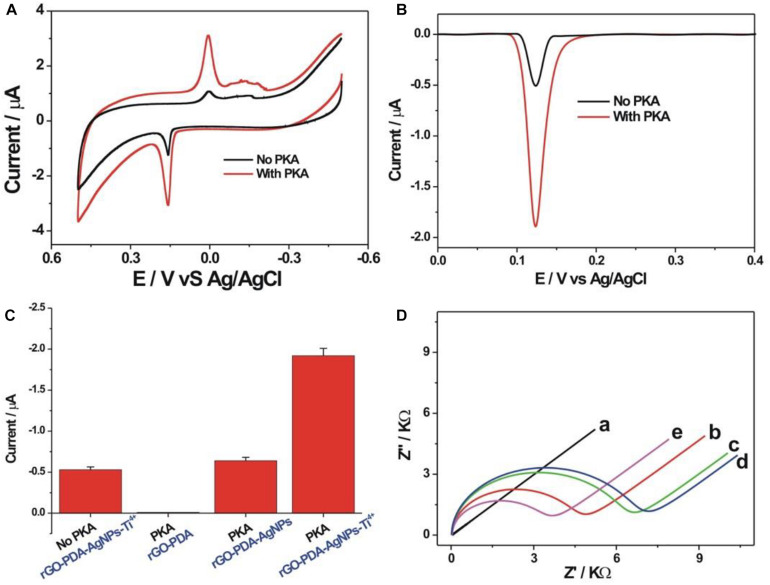
Cyclic voltammetric **(A)** and differential pulse voltammetric **(B)** curves of the PKA biosensor toward blank (black curve) and 10 U/mL PKA (red curve) in 10 mM Tris-HCl (10 mM NaCl, pH 7.4). **(C)** Comparison of DPV responses toward 10 U/mL PKA obtained at different control conditions. The rGO-PDA and rGO-PDA-AgNPs were used as the substitutes of the rGO-PDA-AgNPs-Ti^4+^, respectively. **(D)** Electrochemical impedance spectroscopy for the differently assembled electrodes in 5 mM [Fe(CN)_6_]^3– /4–^ of 10 mM PBS buffer (pH 7.4, 1 M KCl) including the bare gold electrode (a), kemptide-immobilized electrode (b), after MCH blocking (c), phosphorylation of kemptide (d), and recognition with the rGO-PDA-AgNPs-Ti^4+^ nanocomposite (e). EIS was operated with the frequency range from 0.1 Hz to 10 kHz and the amplitude of 5 mV.

The detection feasibility toward PKA activity was verified by CV and DPV measurements. As shown in Figure 3A, a pair of redox peaks related with AgNPs could be seen with the oxidation and reduction potential of about 0.158 and 0 V, respectively, which corresponded to the oxidation of Ag into Ag^+^ and the reduction of Ag^+^ into Ag. In the presence of PKA, the kemptide substrate was phosphorylated for the further binding with the rGO-PDA-AgNPs-Ti^4+^ nanocomposite via the coordination of Ti^4+^ with the phosphate group. The redox peak current of AgNPs in the case of PKA was distinctly larger than that in the case of no PKA. In the absence of PKA, the immobilized kemptide could not be phosphorylated, and thus the rGO-PDA-AgNPs-Ti^4+^ nanocomposite could not effectively bind on the electrode surface. The background response might be attributed to the non-specific adsorption of rGO-PDA-AgNPs-Ti^4+^ nanocomposite onto electrode surface. The oxidation peak current of AgNPs was then quantified by DPV method (Figure 3B). The signal to background ratio toward 10 U/mL PKA was about 3.77. The detection feasibility toward PKA by the rGO-PDA-AgNPs-Ti^4+^ nanocomposite was also verified by the corresponding control experiment with the use of rGO-PDA and rGO-PDA-AgNPs as the substitutes of rGO-PDA-AgNPs-Ti^4+^ (Figure 3C). The rGO-PDA showed no electrochemical response toward the phosphorylated kemptide by PKA since no AgNPs were introduced in the nanocomposite. In the case of rGO-PDA-AgNPs but no Ti^4+^, only a comparable electrochemical response with the background value was observed, indicating that the rGO-PDA-AgNPs could not effectively bind with the phosphorylated kemptide without the aid of Ti^4+^. These control experiments further verified the recognition of rGO-PDA-AgNPs-Ti^4+^ with the phosphorylated kemptide for the electrochemical response of PKA activity. The electrochemical impedance spectroscopy (EIS) was employed to characterize the fabrication process of PKA biosensor (Figure 3D). The bare electrode demonstrated an almost straight line, suggesting the electrochemical process was diffusion-controlled (curve a). The charge transfer resistance (Rct) was increased sequentially to be about 4,843 Ω (curve b) and 6,667 Ω (curve c) after the stepwise assembly of kemptide and MCH. Thus, the immobilized insulating layer prohibited the diffusion of [Fe(CN)_6_]^3–/4–^ toward electrode. The Rct value was observed with a further slight increment to be about 7,054 Ω after PKA-catalyzed kemptide phosphorylation. The possible mechanism might be the incurred negative charge after phosphorylation for the diffusion inhibition of [Fe(CN)_6_]^3–/4–^ (curve d). With the rGO-PDA-AgNPs-Ti^4+^ binding on the electrode via the multi-coordinative interaction between Ti^4+^ and phosphate group, the Rct value was distinctly decreased to be 3,629 Ω (curve e). It could be easily understood that the rGO, PDA and AgNPs collectively afforded an excellent electrical conductivity for the improved response of [Fe(CN)_6_]^3–/4–^ toward electrode surface. The electrochemical impedance characterizations suggested the successful fabrication of the electrochemical PKA biosensor.

### Experimental Condition Optimization

**FIGURE 4:**
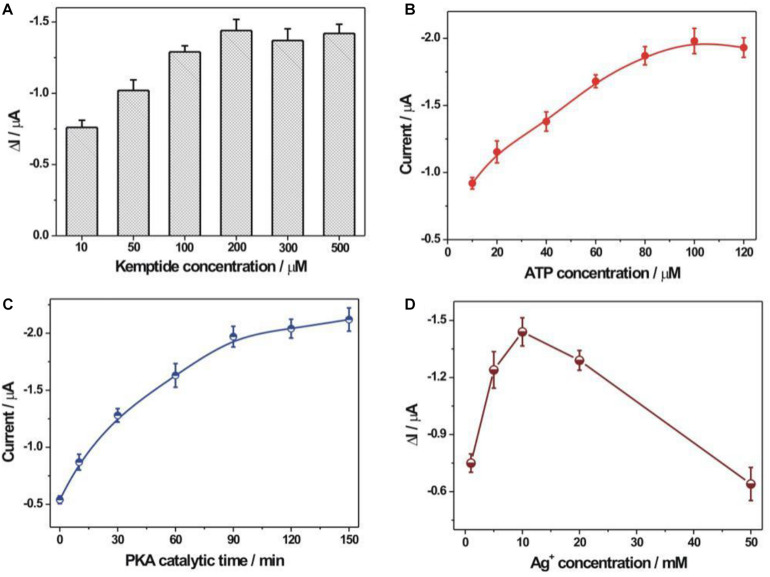
Optimization of experimental conditions. **(A)** The immobilization concentration of kemptide. Various kemptide concentrations (10, 50, 100, 200, 300, and 500 μM) were applied. **(B)** ATP concentration optimization. A series of ATP concentrations including 10, 20, 40, 60, 80, 100, and 120 μM were used. **(C)** The optimization of PKA catalytic time. The employed PKA catalytic time was 0, 10, 30, 60, 90, 120, and 150 min, respectively. **(D)** The optimization of AgNO_3_ concentration during the synthesis of rGO-PDA-AgNPs nanocomposite. Various AgNO_3_ concentration (1, 5, 10, 20, and 50 mM) were studied. The error bars were based on at least three repetitive experiment results. The used PKA concentration was 10 U/mL.

The experimental optimization was performed to pursue the best detection performance (Figure 4). The immobilization concentration of kemptide was first optimized (Figure 4A). It could be seen that the electrochemical response toward PKA increased with the increasing kemptide concentrations from 10 to 200 μM and then slightly decreased at the concentration of kemptide over 200 μM. It might be explained that the increased immobilization amount of kemptide at a higher concentration could by captured by more rGO-PDA-AgNPs-Ti^4+^ nanocomposites for the enhanced electrochemical response. But too large immobilization amount of kemptide onto electrode might be not beneficial for the recognition of rGO-PDA-AgNPs-Ti^4+^ nanocomposites owing to the possible steric hindrance effect. During the PKA catalyzed kemptide phosphorylation process, the phosphate group was originated from the ATP. Thus, the ATP concentration would have an important effect on the PKA catalyzed kemptide phosphorylation process. The ATP concentration was then optimized and shown in Figure 4B. A maximum electrochemical response was achieved at the ATP concentration of 100 μM. The PKA catalytic time was also optimized (Figure 4C). The electrochemical response could almost reach the plateau value at the reaction time of 90 min and the increasing tendency became slowly after 90 min. Thus the optimized time for PKA catalyzed phosphorylation process was chosen as 90 min. The Ag^+^ concentration during the synthesis of rGO-PDA-AgNPs-Ti^4+^ nanocomposites was also optimized (Figure 4D). In theory, more amounts of Ag^+^ could increase the loading amount of AgNPs onto PDA layer for the increased signal response. However, too much AgNPs onto PDA layer would restrict the chelation of Ti^4+^ cations with the diol groups of PDA and then its recognition ability with the phosphorylated kemptide for the limited detection performance. It could be seen that the rGO-PDA-AgNPs nanocomposites prepared by using Ag^+^ concentration of 10 mM could achieve the best electrochemical response toward PKA activity.

### Detection Performance for PKA Activity

**FIGURE 5:**
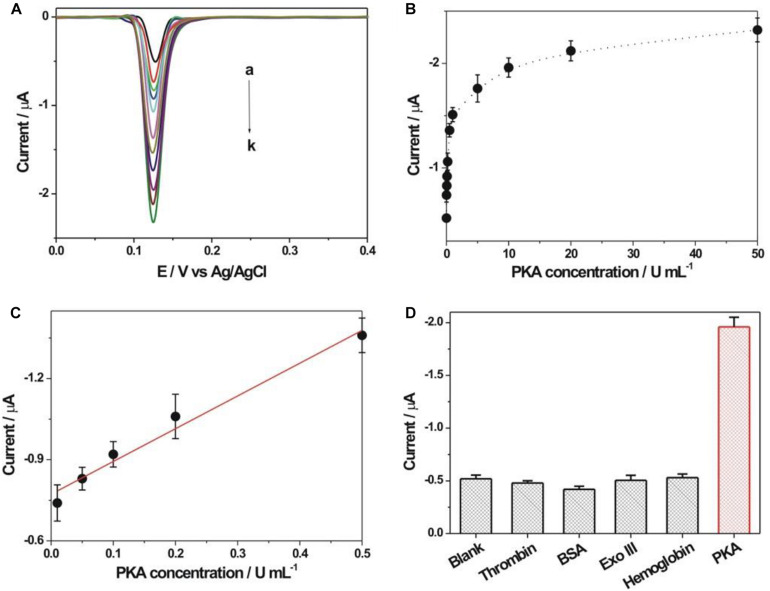
**(A)** DPV responses obtained at different concentrations of PKA. The PKA concentrations from curve a to k were 0, 0.01, 0.05, 0.1, 0.2, 0.5, 1, 5, 10, 20, and 50 U/mL, respectively. **(B)** Calibration curve of DPV response vs. PKA concentration. **(C)** The linear plot between DPV peak current and PKA concentration. **(D)** Electrochemical response comparison toward thrombin (1 μM), bovine serum albumin (BSA, 1 μM), Exo III (50 U/mL), hemoglobin (1 mg/mL) and PKA (10 U/mL), respectively.

As shown in Figure 5A, the detection performance of the fabricated biosensor toward PKA activity was investigated. The DPV response increased accordingly with the increment of PKA concentration (0–50 U/mL). The calibration curve for the peak current vs. PKA concentration was shown in Figure 5B. The linear relationship between the DPV response and the PKA concentration in the range of 0.01–0.5 U/mL was obtained (Figure 5C). The linear regression equation was listed as Y (peak current, μA) = −0.7748–1.206 X (concentration, U/mL) with a regression coefficient of 0.9906. The detection limit toward PKA could be experimentally achieved as 0.01 U/mL, which was evidently lower than many reported methods (Supplementary Table S1). Thus, the fabricated PKA biosensor could achieve the sensitive detection toward PKA activity. The detection reproducibility of the fabricated biosensor toward PKA was studied. Five repetitive measurements toward two concentrations of PKA (0.5 and 10 U/mL) displayed the relative standard deviations (RSDs) of 5.3 and 4.9%, respectively, suggesting the satisfied detection reproducibility toward PKA. The selectivity experiment of the fabricated PKA biosensor was studied by using several different proteins (Figure 5D). Some non-specific proteins including thrombin (1 μM), bovine serum albumin (BSA, 1 μM), Exo III (50 U/mL), hemoglobin (1 mg/mL) and PKA (10 U/mL) were employed as comparison. The response currents for these non-specific proteins were basically comparable with that of the blank sample, indicating the good detection selectivity toward PKA. The stability of the kemptide-immobilized electrode was checked by storage in the refrigerator (4^*o*^C) for 1 week. The average electrochemical response of the kemptide-immobilized electrode based on three repetitive experiments toward 10 U/mL PKA was about 96.4% of the initial electrochemical response (Supplementary Figure S2), indicating the relatively well stability of the kemptide-immobilized electrode.

### Applicative Potential and Inhibitor Screening of the Fabricated Biosensor

**FIGURE 6:**
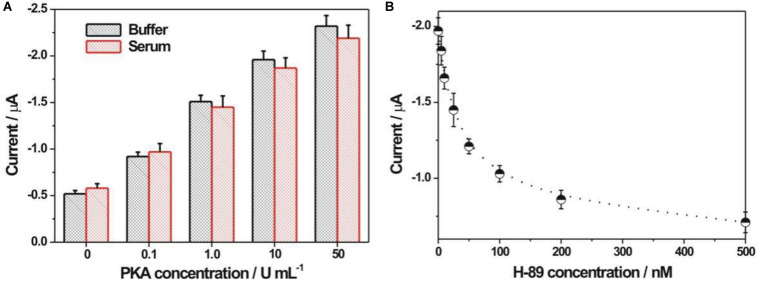
**(A)** Electrochemical detection of PKA in buffer and 5% diluted FBS. **(B)** Electrochemical responses at different H-89 concentrations. The H-89 concentrations were 5, 10, 25, 50, 100, 200, and 500 nM, respectively. The concentrations for PKA and ATP were 10 U/mL and 100 μM, respectively.

The applicative feasibility of the PKA biosensor in the diluted serum samples was further studied (Figure 6A). Both the electrochemical responses toward PKA in the diluted serum and in the buffer were basically consistent. Meanwhile, the electrochemical signal responded increasingly upon the increment of PKA concentration in the serum, suggesting the applicative potential in the complex biological samples. The recovery experiments toward two spiked concentrations of PKA (0.2 and 0.4 U/mL) in the diluted serum were conducted with an acceptable recovery ratio of about 119 and 92.5%, respectively (Supplementary Table S2). To verify if current PKA biosensor could be used for inhibitor screening, H-89 was chosen as a model inhibitor since it had been well-recognized as an efficient PKA inhibitor. It could be seen from Figure 6B that an evident inhibition effect of the H-89 toward PKA activity could be observed. The calculated IC_50_ value, corresponding to the inhibitor concentrations that could inhibit the enzyme activity by 50%, was 48 nM, which was basically consistent with the reported value (MacConaghy et al., 2014; Chen et al., 2018). It thus demonstrated that the current electrochemical sensing strategy could be used for PKA inhibitor evaluation.

## Conclusion

In this study, a novel rGO-PDA-AgNPs-Ti^4+^ nanocomposite was successfully prepared, which was used as an integral recognition and signaling element to fabricate electrochemical sensing platform for protein kinase activity and inhibitor assay. During the preparation of rGO-PDA-AgNPs-Ti^4+^ nanocomposite, the PDA layer provided the binding sites for Ag^+^ and Ti^4+^ adsorption. Also, the Ag^+^ was *in situ* reduced into AgNPs. The phosphorylated kemptide by PKA onto electrode could be specifically recognized by rGO-PDA-AgNPs-Ti^4+^ nanocomposite based on the multicoordinative interaction between Ti^4+^ and the phosphate group. The AgNPs onto nanocomposite contributed to the direct and amplified electrochemical signal readout related with PKA activity. The developed PKA biosensor based on the rGO-PDA-AgNPs-Ti^4+^ nanocomposite could be easily operated with no need of expensive or specialized reagents or complicate post-treatment steps. The detection limit for PKA was obtained as 0.01 U/mL, which was lower than most of the reported methods. It could be also extended for inhibitor screening. The current biosensor by rGO-PDA-AgNPs-Ti^4+^ nanocomposite is promising as a versatile means for the assay of protein kinase activity, and might hold the potential in disease diagnosis, prognosis, and drug discovery.

## Data Availability Statement

The original contributions presented in the study are included in the article/Supplementary Material, further inquiries can be directed to the corresponding authors.

## Author Contributions

JW and SL designed the work and wrote the manuscript. JW, XL, CW, DL, and LW carried out the experiments. JW, CW, and FL performed the statistical analysis. FL and SL revised and edited the manuscript. All authors reviewed the manuscript and have agreed to its publication.

## Conflict of Interest

The authors declare that the research was conducted in the absence of any commercial or financial relationships that could be construed as a potential conflict of interest.
