# Supplementary material for: An Integral Recognition and Signaling for Electrochemical Assay of Protein Kinase Activity and Inhibitor by Reduced Graphene Oxide-Polydopamine-Silver Nanoparticle-Ti4+ Nanocomposite
Source: Front Bioeng Biotechnol. 2020 Nov 13;8:603083. doi: 10.3389/fbioe.2020.603083 (PMC7691532; doi:10.3389/fbioe.2020.603083)
Supplement: Supplementary file 1 [file Table_1.DOCX]

Supplementary Material


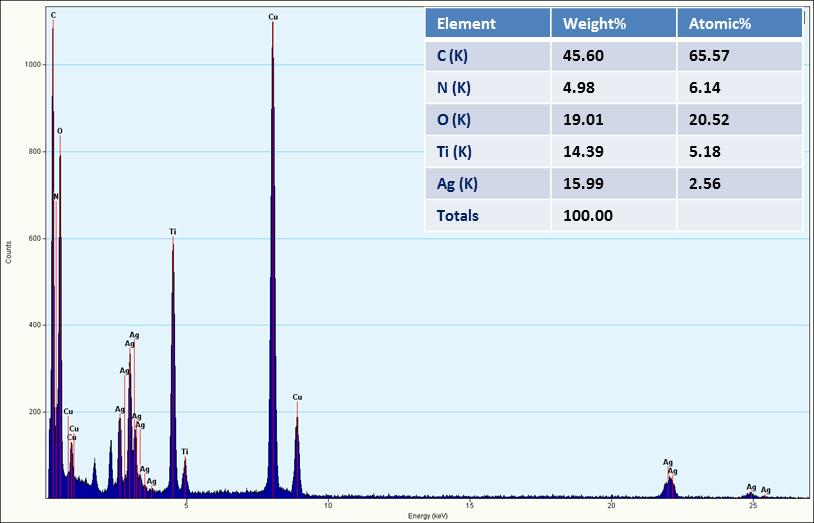


**Figure S1.** The EDX spectra of the rGO-PDA-AgNPs-Ti^4+^ nanocomposite. Inset shows the corresponding contents of C, N, O, Ag and Ti in rGO-PDA-AgNPs-Ti^4+^ nanocomposite.


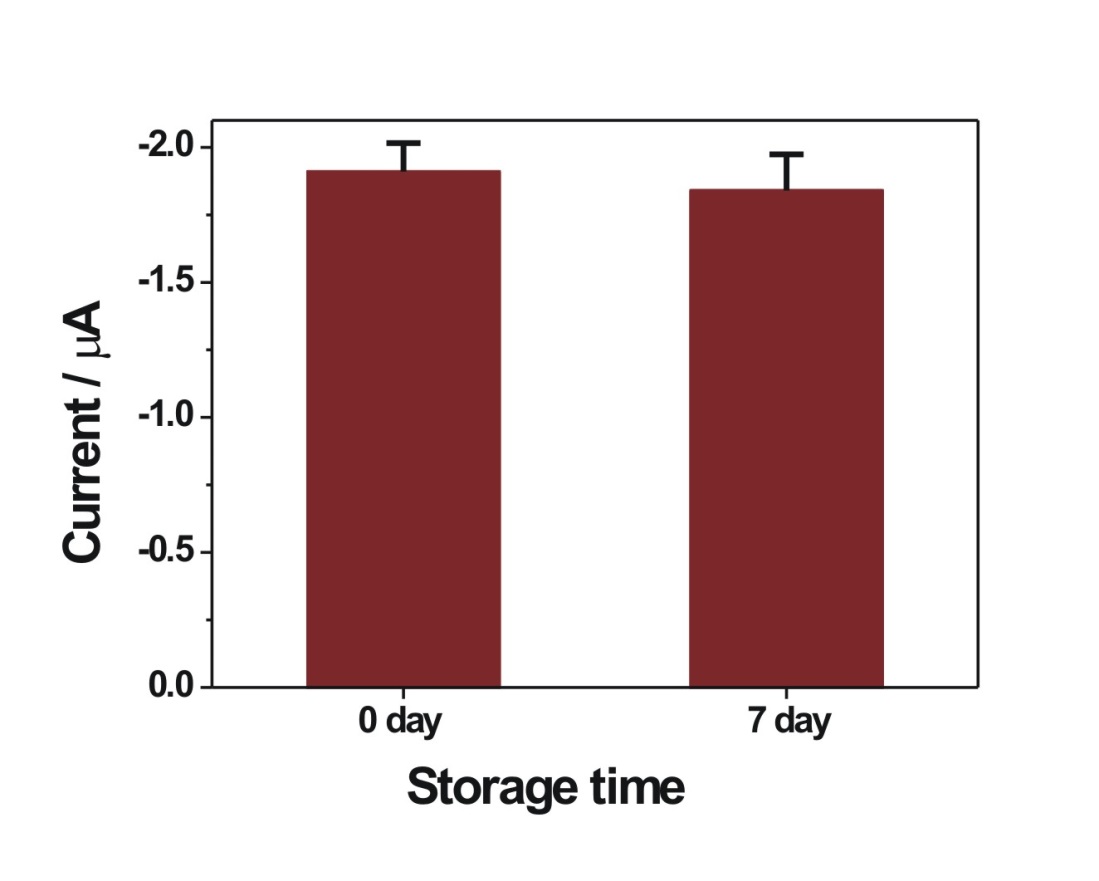


**Figure S2.** The storage stability of the kemptide modified electrode. The electrochemical response toward 10 U/mL PKA was obtained after the storage of the kemptide modified electrode for different time (0 and 7 days). The error bars were obtained based on three repetitive experiments.

**Table S1.** Detection performance compassion of current method with these reported methods.

| **Method** | **Strategy** | **Linear range**  **(U mL^-1^)** | **Detection limit**  **(U mL^-1^)** | **Ref.** |
| --- | --- | --- | --- | --- |
| Electrochemistry | Zr^4+^-DNA-AuNPs | 0.1**-** 40 | 0.03 | 1 |
| Electrochemistry | Phos-tag and enzymatic amplification | 0.5 **-** 25 | 0.15 | 2 |
| Electrochemistry | Zr^4+^ and RCA | 5 **-** 500 | 0.5 | 3 |
| Electrochemistry | Zr^4+^-DNA-AuNPs | - | 0.15 | 4 |
| Electrochemistry | TiO2-assisted silver nanoparticle | 0.2 - 1 | 0.2 | 5 |
| Electrochemistry | eATRP | 0 **-** 0.14 | 0.00163 | 6 |
| Electrochemistry | Carboxypeptidase Y-assisted peptide cleavage | 0.1 - 50 | 0.083 | 7 |
| Electrochemistry | AuNPs/MWNTs nanohybrids | 0.1 **-** 1 | 0.09 | 8 |
| Electrochemistry | rGO-PDA-AgNPs-Ti^4+^ nanocomposite | 0.01 **-** 0.5 | 0.01 | This  work |

**Table S2.** Recovery experiments of PKA in diluted fetal calf serum

| **Samples** | **Added (U/mL)** | **Detected (U/mL)** | **Recovery (%)** | **RSD (%)** |
| --- | --- | --- | --- | --- |
| 1 | 0.2 | 0.238 | 119 | 7.3 |
| 2 | 0.4 | 0.37 | 92.5 | 5.8 |

RSD was obtained based on three repetitive experiments. The recovery ratio was obtained with the division of the detected value by the added value.

**References:**

(1) Wang, Z., Sun, N., He, Y., Liu, Y., and Li, J. (2014). DNA assembled gold nanoparticles polymeric network blocks modular highly sensitive electrochemical biosensors for protein kinase activity analysis and inhibition. *Anal. Chem.* 86, 6153-6159. doi: 10.1021/ac501375s.

(2) Yin, H., Wang, M., Li, B., Yang, Z., Zhou, Y., and Ai, S. (2015). A sensitive electrochemical biosensor for detection of protein kinase A activity and inhibitors based on phos-tag and enzymatic signal amplification. *Biosens. Bioelectron.* 63, 26-32. doi: 10.1016/j.bios.2014.07.016.

(3) Miao, P., Ning, L., Li, X., Li, P., and Li, G. (2012). Electrochemical strategy for sensing protein phosphorylation. *Bioconjugate Chem.* 23, 141-145. doi: 10.1021/bc200523p.

(4) Xu, X., Nie, Z., Chen, J., Fu, Y., Li, W., Shen, Q., and Yao, S. (2009). A DNA-based electrochemical strategy for label-free monitoring the activity and inhibition of protein kinase. *Chem. Commun.* 6946-6948. doi: 10.1039/b913943e.

(5) Ji, J., Yang, H., Liu, Y., Chen, H., Kong, J., and Liu, B. (2009). TiO_2_-assisted silver enhanced biosensor for kinase activity profiling. *Chem. Commun.* 1508-1510. doi: 10.1039/b820738k.

(6) Hu, Q., Wang, Q., Jiang, C., Zhang, J., Kong, J., and Zhang, X. (2018). Electrochemically mediated polymerization for highly sensitive detection of protein kinase activity. *Biosens. Bioelectron.* 110, 52-57. doi: 10.1016/j.bios.2018.03.030.

(7) Liu, J., Cheng, H., He, D., He, X., Wang, K., Liu, Q., Zhao, S., and Yang, X. (2017). Label-free homogeneous electrochemical sensing platform for protein kinase assay based on carboxypeptidase Y-assisted peptide cleavage and vertically ordered mesoporous silica films. *Anal. Chem.* 89, 9062-9068. doi: 10.1021/acs.analchem.7b01739.

(8) Liu, J., He, X., Wang, K., Wang, Y., Yan, G., and Mao, Y. (2014). Amplified electrochemical detection of protein kinase activity based on gold nanoparticles/multi-walled carbon nanotubes nanohybrids. *Talanta* 129, 328-335. doi: 10.1016/j.talanta.2014.05.043.
